# Immunosenescence and Inflammaging as Drivers of Neurodegeneration: Cellular Mechanisms, Neuroimmune Crosstalk, and Therapeutic Implications

**DOI:** 10.3390/cells15080657

**Published:** 2026-04-08

**Authors:** Gianmarco Bertoni, Sara Ristori, Daniela Monti

**Affiliations:** Department of Experimental and Clinical Biomedical Sciences “Mario Serio”, University of Florence, 50134 Florence, Italy; gianmarco.bertoni@unifi.it (G.B.); sara.ristori@unifi.it (S.R.)

**Keywords:** immunosenescence, aging, inflammaging, neuroinflammation, neurodegenerative diseases, geroscience, senolytics, immunomodulation, therapeutic strategies

## Abstract

**Highlights:**

**What are the main findings?**

- Aging-associated immunosenescence and inflammaging drive neurodegeneration by impairing microglial and astrocyte function and disrupting systemic–CNS immune crosstalk.
- Both innate and adaptive immune compartments contribute to sustained neuroinflammation, synaptic dysfunction, and neuronal loss.

**What are the implications of the main findings?**

- Targeting senescent cells, restoring immune balance, and modulating inflammation may offer therapeutic avenues for neurodegenerative diseases.
- Early intervention in immune aging could enhance brain resilience and slow disease progression.

**Abstract:**

Aging is accompanied by profound alterations in immune function, termed immunosenescence, and by a chronic, low-grade inflammatory state known as inflammaging. These processes are increasingly recognized as central drivers of age-related neurodegenerative diseases, including Alzheimer’s Disease, Parkinson’s Disease, Amyotrophic Lateral Sclerosis and Multiple Sclerosis. In the central nervous system, senescent microglia and astrocytes lose their homeostatic and neuroprotective functions, while systemic immune aging and blood–brain barrier dysfunction further amplify neuroinflammation and impair protein aggregate clearance. This sustained pro-inflammatory environment promotes synaptic dysfunction, neuronal loss and cognitive decline. Here, we synthesize current knowledge of the mechanistic links among immunosenescence, inflammaging, and neurodegeneration, highlighting innate and adaptive immune dysregulation, mitochondrial impairment, and failed resolution pathways. We further discuss emerging therapeutic strategies, including senolytics, immunoceuticals, microbiome-based interventions and advanced drug delivery systems, aimed at restoring immune homeostasis and enhancing brain resilience. By integrating mechanistic and translational insights, this review provides a framework for developing novel interventions to target immune aging in neurodegenerative diseases.

## 1. Introduction

Aging is a systemic, progressive biological remodeling process that reshapes tissue homeostasis throughout the lifespan [1]. Immunosenescence, together with chronic low-grade inflammation known as inflammaging, reflects the age-associated decline in immune competence, characterized by coordinated functional, structural and metabolic alterations rather than a sudden failure [2]. These changes include remodeling of lymphoid tissues, shifts in immune cell composition and dysregulation of immune responses, ultimately reducing the ability to respond to novel pathogens [2,3]. Immunosenescence is highly heterogeneous and influenced by genetic, environmental, lifestyle and nutritional factors, which collectively shape individual immune aging trajectories and disease susceptibility [4,5,6,7,8].

As a consequence, older adults are more susceptible to infections, autoimmunity, cancer and neurodegenerative diseases (NDDs). NDDs represent a major challenge of population aging due to their rising prevalence, inter-individual variability and the lack of disease-modifying therapies. These disorders are characterized by the gradual loss of neurons, which progressively impairs motor, sensory and cognitive functions [2,9].

Growing evidence suggests that immunosenescence and inflammaging are not merely secondary consequences of neurodegeneration but actively contribute to disease susceptibility, progression and therapeutic resistance. Systemic immune aging and immune dysfunction within the central nervous system (CNS) converge to establish a persistent pro-inflammatory milieu that may disrupt neuronal homeostasis and contribute to neurodegeneration [10]. Emerging data also indicate that age-related alterations in peripheral immunity can influence neuroimmune crosstalk and may modulate disease onset and progression [9,11,12].

This review integrates current knowledge of immunosenescence and inflammaging with mechanistic insights into major NDDs, including Alzheimer’s disease (AD), Parkinson’s disease (PD), Amyotrophic Lateral Sclerosis (ALS), and Multiple Sclerosis (MS), highlighting both shared immune aging signatures and disease-specific trajectories. In addition, it critically examines emerging therapeutic strategies targeting immune dysfunction, such as senotherapeutics, immune rejuvenation approaches, metabolic and microbiota-based interventions, and advanced drug-delivery systems, highlighting their translational potential and current limitations. Overall, the review frames immunosenescence and inflammaging as central biological processes linking systemic aging to CNS degeneration and as promising therapeutic targets for extending healthspan rather than merely lifespan.

Although substantial evidence supports a key role for immunosenescence and inflammaging in neurodegeneration, current data increasingly suggest a bidirectional and self-reinforcing relationship rather than a strictly unidirectional causal pathway [13,14].

## 2. Immunosenescence and Inflammaging: Conceptual Framework and Systemic Implications

Immunosenescence is the progressive remodeling of the immune system associated with aging, resulting in impaired immune surveillance, reduced responsiveness to novel pathogens and vaccines, and increased susceptibility to cancer and chronic inflammatory diseases [15]. Hallmarks of immunosenescence include thymic involution, reduced naïve T cells output and T cell receptor (TCR) repertoire, accumulation of memory and terminally differentiated lymphocytes, diminished innate immune function and compromised coordination between innate and adaptive immune responses [15]. Age-related hematopoietic stem cell skewing toward the myeloid lineage and dysfunction of monocytes, macrophages, and dendritic cells further contribute to a chronic pro-inflammatory state [16].

A central consequence of immunosenescence is inflammaging, a persistent, sterile low-grade inflammation driven by senescent cell accumulation, chronic exposure to damage-associated molecular patterns (DAMPs), alterations in the microbiota, and defective clearance mechanisms [17,18,19,20,21,22,23,24]. Cellular senescence acts as a mechanistic bridge between immunosenescence and inflammaging: impaired immune clearance allows senescent cells to accumulate, while their SASP factors amplify systemic inflammation through pathways such as NLRP3 inflammasome activation and cGAS–STING signaling, thereby establishing a self-reinforcing inflammatory loop [19,20,25,26,27,28,29,30,31,32,33,34,35,36,37].

Additionally, metabolic rewiring of aged immune cells, including mTOR hyperactivation, mitochondrial dysfunction, altered NAD+ homeostasis, and disrupted AMPK signaling, contributes to immunometabolic dysfunction and reduced immune resilience.

**Figure 1:**
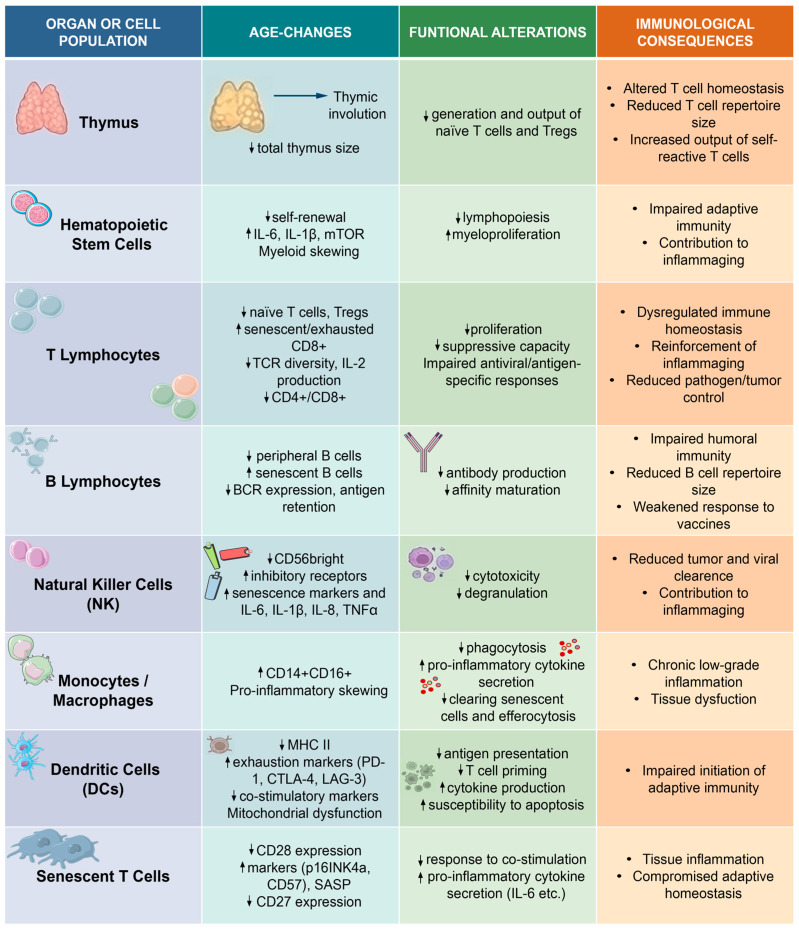
Principal cellular and functional changes in immunosenescence. The figure provides a comprehensive overview of the age-related remodeling of the immune system. It highlights primary thymic involution, leading to a diminished output of naïve T cells, alongside myeloid bias and functional exhaustion of Hematopoietic Stem Cells (HSCs). These central defects, combined with the phenotypic shift of peripheral T and B lymphocytes toward senescent and exhausted states, result in a constricted immune repertoire and impaired response to novel antigens. Furthermore, the functional decline of innate immune cells, such as NK cells, monocytes, macrophages, and dendritic cells (DCs), contributes to chronic, low-grade systemic inflammation (inflammaging) and reduced surveillance against pathogens and malignancies. ↑ indicates “increase” or “upregulation”; ↓ indicates “decrease” or “downregulation”. The references corresponding to the studies originally reported in Figure 1 are indicated here: Row 1 [15,29,38,39]; Row 2 [40,41,42,43,44]; Row 3 [45,46,47,48,49,50,51,52,53,54,55,56,57,58,59]; Row 4 [60,61,62,63,64,65,66]; Row 5 [67,68,69,70,71]; Row 6 [41,42,72,73,74]; Row 7 [75,76,77]; Row 8 [78,79,80].

The principal cellular and functional alterations of immunosenescence are summarized in Figure 1.

Beyond these classical features, immunosenescence is now recognized as a multifactorial systemic process involving interconnected hallmarks such as genomic instability, telomere attrition, epigenetic dysregulation, loss of proteostasis, deregulated nutrient sensing, mitochondrial dysfunction, stem cell exhaustion, altered intercellular communication, microbiome dysbiosis, cellular senescence and chronic low-grade inflammation (inflammaging) [9]. Together, these hallmarks converge to impair immune cell fitness, regenerative capacity, and signaling fidelity, thereby reshaping immune function across tissues and throughout the lifespan.

Importantly, immunosenescence and inflammaging reflect biological rather than chronological aging and are shaped by cumulative stressors and chronic infections [81,82,83,84,85]. They ultimately represent a decline in immune resilience and the capacity to restore homeostasis following stress, providing a key framework for geroscience-based interventions.

A critical distinction must be made between physiological immune aging and disease-associated immune dysregulation. During physiological aging, immunosenescence and inflammaging represent adaptive remodeling processes characterized by low-grade inflammation and reduced immune responsiveness. In contrast, neurodegenerative diseases involve a pathological amplification of these mechanisms, with exaggerated inflammatory signaling, impaired resolution pathways and disease-specific triggers such as protein aggregation (e.g., Aβ, Tau, α-synuclein). Thus, while immunosenescence provides a permissive background, disease-specific factors drive the transition from physiological aging to pathological neurodegeneration [18,86,87].

### 2.1. Interplay Between Immunosenescence and Inflammaging: A Self-Reinforcing Loop

Building on the conceptual framework outlined above, this section focuses on the mechanistic circuits that sustain the immunosenescence–inflammaging axis across immune compartments.

SASP-derived inflammatory mediators impair hematopoietic stem and progenitor cell function, limiting immune reconstitution and the generation of naïve lymphocytes. Concurrently, chronic inflammatory signaling promotes the expansion of immunosuppressive or dysfunctional immune populations, including regulatory T cells, myeloid-derived suppressor cells, and M2-like macrophages. This expansion is driven by persistent exposure to pro-inflammatory cytokines (e.g., IL-6 and TGF-β) and metabolic stress signals, which trigger compensatory mechanisms initially aimed at limiting collateral tissue damage. However, in the context of aging, this adaptive response becomes maladaptive, leading to a state of ‘immune paralysis’ that further weakens adaptive immunity, impairs effective immune surveillance, and hinders tissue repair [3,88,89,90]. This altered immune landscape promotes inflammation without effective pathogen clearance.

Age-related impairments in antigen presentation, pathogen sensing, and adaptive responses lead to persistent microbial and cellular stress signals that continuously activate innate immune cells and drive the production of pro-inflammatory cytokines, including IL-6, TNF-α, and IL-1β [8,80], thereby reinforcing systemic and tissue-specific inflammation [6]. This paradoxical coexistence of chronic immune activation and functional deficiency is a defining feature of immune aging.

At the organismal level, systemic compartments exhibit persistent inflammatory signaling, while local tissues display dysregulated immune responses that disrupt metabolic and structural homeostasis without eliciting an effective adaptive immune response [7,8]. These processes establish a self-amplifying loop in which inflammation impairs immune regeneration, defective immunity sustains unresolved stress signals, and tissue damage perpetuates inflammatory output.

Peripheral immune aging also influences brain structure and function by altering cytokine profiles, immune cell trafficking and blood–brain barrier (BBB) permeability [8,9,30,76,91]. In parallel, CNS immune cells undergo aging-associated changes that increase vulnerability to neurodegeneration [9]. This bidirectional immune-brain interaction provides a conceptual link among systemic immune aging, neuroinflammatory and neurodegenerative processes.

### 2.2. The Mechanistic Interplay Between Immunosenescence and Neurodegeneration

Profound bidirectional interactions between the immune system and the CNS emerge during aging. Immune remodeling occurring with advancing age in both peripheral and central compartments contributes to the establishment of a chronic pro-inflammatory environment, which is associated with progressive impairment of neuronal function, synaptic plasticity and tissue homeostasis. This inflammatory state in the aging brain, commonly referred to as neuroinflammaging, arises from the convergence of immunosenescence, systemic inflammaging and dysfunction of intrinsic CNS immune cells, potentially increasing susceptibility to neurodegenerative disease [92,93].

Immunosenescence affects both innate and adaptive immune responses, leading to an imbalance between pro- and anti-inflammatory signaling pathways. Reduced immune surveillance, impaired pathogen clearance and defective resolution of inflammation sustain a state of chronic immune activation. Importantly, these systemic immune alterations propagate to the CNS through circulating cytokines, immune cell trafficking and age-associated BBB dysfunction, thereby contributing to reshaping the neuroimmune environment and potentially accelerating neurodegenerative processes [9,18,30]. Moreover, persistent neuroinflammation is associated with impaired brain regenerative capacity by depleting neural stem cells (NSCs) and inhibiting their differentiation into functional neurons [94,95]. Within the CNS, additional age-related changes, such as microglial priming, astrocytic senescence, and dysregulation of the complement system, further lower the threshold for neurodegenerative cascades. In particular, metabolic reprogramming of microglia during aging, characterized by increased glycolytic dependence and mitochondrial dysfunction, amplifies pro-inflammatory signaling while reducing phagocytic efficiency and debris clearance [96]. Together, these interconnected mechanisms create a permissive environment for protein aggregation, synaptic dysfunction and progressive neuronal loss.

## 3. Innate Immune Dysregulation in the Aging Brain: Glial Senescence and Blood–Brain Barrier Dysfunction

### 3.1. Glial Senescence and Innate Immune Remodeling in the Aging Brain

The innate immune system undergoes progressive functional decline with aging, affecting macrophages, dendritic cells, granulocytes, natural killer (NK) cells, and CNS-resident microglia and astrocytes [10,97]. Within the CNS, microglia and astrocytes exhibit profound age-related remodeling that disrupts neuronal homeostasis and tissue surveillance.

Microglia show mitochondrial dysfunction-driven oxidative stress [98], impaired phagocytosis [99], and extensive transcriptomic remodeling [100,101]. A balance between environmental sensing and genomic integrity governs the functional transition of microglia during aging. Triggering Receptor Expressed on Myeloid cells 2 (TREM2), a key surface receptor involved in lipid sensing and the clearance of protein aggregates, plays a pivotal role in this process. Notably, the expansion of microglial subpopulations expressing high levels of TREM2 has been associated with chronic neuroinflammation and cognitive impairment during aging [102]. In parallel, the maintenance of the microglial genome is equally critical. For example, deficiency of Excision Repair 1, non-catalytic subunit (ERCC1), a key component of the nucleotide excision repair pathway, triggers intense genotoxic stress, which in turn accelerates the onset of microglial senescence [5,103].

Defective autophagy and mitophagy further drive microglial senescence, promoting organelle accumulation, reduced proliferative capacity, CDKN1A/p21 upregulation, dystrophic morphology and enhanced SASP release [104]. Mitochondrial dysfunction is exacerbated in neurodegenerative contexts such as AD, where amyloid-β (Aβ) burden amplifies oxidative stress and senescence [105]. Release of mitochondrial DNA activates cGAS–STING signaling, sustaining chronic inflammation and neurodegeneration [30], thereby linking metabolic stress to innate immune activation, suggesting a feed-forward loop between mitochondrial dysfunction and inflammatory signaling.

The gut microbiota has emerged as an important upstream regulator of microglial aging. Microbiota-derived signals are required for physiological microglial maturation, as germ-free or antibiotic-treated mice display transcriptional and functional impairments in microglia [106,107]. Age-associated dysbiosis promotes oxidative stress, mitochondrial dysfunction and senescence-like microglial phenotypes, while microbiota depletion attenuates these defects [98,108].

Increased intestinal permeability and systemic inflammation elevate circulating TNF and IL-6 [109], modulating microglial activation and amyloidosis in a sex-specific manner, further suggesting a regulatory role for gut microbiota in regulating CNS immune cell function [107]. Thus, peripheral microbial changes are increasingly recognized to shape CNS immune aging.

Senescent microglia display a senescence-like phenotype: p16INK4A upregulation, cell cycle arrest, altered chromatin, lipid droplet accumulation, defective phagocytosis, excessive ROS production and SASP secretion [110,111], thereby shifting from homeostatic sentinels to chronically primed inflammatory effectors. Impaired CD36 signaling reduces the uptake of cellular debris [112], while aberrant CD22-mediated signaling further inhibits the clearance of myelin debris, Aβ and α-synuclein aggregates [113]. Additional contributors include progranulin deficiency [111,114], dysregulated ATP–P2X7 signaling [115], reduced RICTOR expression [116], lipid peroxidation and ferroptosis-related pathways [117] and replicative stress [118], collectively increasing susceptibility to NDDs [119,120,121].

Astrocytes also undergo senescence-associated remodeling. Aging astrocytes exhibit proteostasis loss, lysosomal and proteasomal dysfunction, impaired synaptogenic support and autophagosome accumulation [122]. Alterations in IL-10 receptor signaling and cholesterol biosynthesis impair microglial homeostasis during innate immune activation [123]. These alterations underscore the interdependence between astrocytic senescence and microglial dysfunction.

Reduced lamin B1 contributes to nuclear instability and altered gene regulation [124], while oxidative stress, proteasome inhibition, replicative exhaustion and pathological proteins induce astrocyte senescence [125]. In AD, senescent astrocytes induce excitotoxic neuronal death [126]. Aβ, Tau oligomers, IL-1β and oxidative stress propagate senescence via SASP-mediated paracrine signaling [127,128]. Astrocyte function and their ability to support neuronal activity are tightly regulated by conserved signaling pathways that control cellular homeostasis and lifespan. A central component is the Hippo signaling pathway and its primary downstream effector, Yes-associated protein (YAP). As a transcriptional co-activator, YAP supports astrocyte survival, proliferation, and homeostatic functions. Age-related YAP inactivation disrupts these fundamental processes, contributing to astrocyte senescence and reduced neuroprotective capacity in aging and neurodegeneration [129].

Aging also biases astrocytes toward different phenotypes. Within this framework, astrocytes have been broadly categorized into A1-like and A2-like states. Microglia-derived IL-1α, TNF-α and C1q induce a neurotoxic A1-like state characterized by complement activation (e.g., C3 upregulation), inflammatory mediator release and loss of homeostatic support [130,131,132], which in turn perpetuates inflammation by releasing IL-1α, IL-1β, IL-6, IL-10, TNF-α and IL-17, thereby exacerbating neuronal and oligodendrocytic damage and cognitive impairment [133,134].

In contrast, A2-like astrocytes are typically induced by ischemic or reparative stimuli and display neuroprotective and pro-reparative programs. These states are associated with the release of neurotrophic and anti-inflammatory factors that support neuronal survival, tissue repair, angiogenesis, BBB restoration and the resolution of inflammation.

Although the A1/A2 polarization framework does not fully capture the heterogeneity and plasticity of reactive astrocytes, which are now recognized as existing along a dynamic continuum shaped by disease stage and microenvironmental cues, it remains a useful conceptual model for interpreting astrocyte-driven neuroinflammation. Notably, aging biases astrocytes towards sustained pro-inflammatory, A1-like programmes, consistent with a chronic neuroinflammatory milieu. This age-dependent shift likely overlaps with systemic and central immunosenescence-driven remodeling, suggesting that astrocytic polarization both reflects and amplifies immunosenescence and inflammaging within the CNS.

Beyond glial senescence, aging also compromises the integrity of the BBB, further amplifying neuroimmune interactions between the periphery and the CNS.

### 3.2. Blood–Brain Barrier Dysfunction and Neuroimmune Crosstalk

The BBB is a key interface between the peripheral immune system and the CNS. Composed of endothelial cells connected by tight junctions, pericytes, astrocytic endfeet, and extracellular matrix components, the BBB regulates molecular and cellular trafficking while preserving brain homeostasis [135,136,137]. Ageing disrupts the BBB’s structure and function, increasing permeability and impairing neuroimmune communication [138,139,140].

BBB deterioration occurs even in cognitively healthy aging and exacerbates cognitive decline [138]. Structural changes include tight junction disruption, altered transcytosis, basement membrane thickening, endothelial mitochondrial dysfunction, and alterations in the neurovascular unit [141,142,143,144,145]. Age-related pericyte loss further contributes to BBB instability by weakening endothelial support and impairing vascular homeostasis [141,142,143,144,145,146,147,148]. These changes may permit the entry of peripheral cytokines, chemokines and immune cells into the brain parenchyma, linking systemic immune status to CNS inflammation [149].

BBB cells actively participate in immune signaling. Endothelial cells express pattern recognition receptors, including Toll-like receptors (TLRs), which are regulated by oxidative stress and TNF-α [150], while cannabinoid type 2 receptor signaling modulates inflammatory responses, reduces barrier disruption and inhibits macrophage migration [151]. Cytokines such as IL-1β, IL-6, TGF-β2, and ciliary neurotrophic factor can cross the BBB via regulated transport, influencing neuronal and synaptic function [143,152].

Aging further impairs amyloid β clearance by downregulating BBB efflux transporters, such as P-glycoprotein (P-gp) and low-density lipoprotein receptor-related protein 1 (LRP1), and by upregulating the influx receptor for advanced glycation end-products (RAGE), thereby potentially increasing brain uptake of circulating Aβ [153]. Upregulation of leukocyte adhesion molecules enhances immune cell infiltration, amplifying neuroinflammation and cognitive decline [8,154,155]. Microglial activation, astrocytic endfeet disruption, and pericyte loss synergistically impair BBB integrity, establishing a feed-forward loop between immune aging, barrier dysfunction and neurodegeneration [140,142,155,156]. Thus, BBB breakdown is increasingly recognized as both a consequence and an amplifier of immunosenescence-driven neuroinflammatory remodeling.

Importantly, beyond mechanistic insights, emerging human studies provide quantitative support for the relationship between peripheral inflammaging and CNS outcomes. Elevated systemic inflammatory markers, particularly IL-6, TNF-α and C-reactive protein (CRP), have been consistently associated with accelerated cognitive decline and increased risk of dementia. Longitudinal cohort studies indicate that higher midlife IL-6 and CRP levels predict greater cognitive deterioration and hippocampal atrophy later in life. Moreover, meta-analyses have shown that circulating inflammatory biomarkers correlate with neurodegenerative hallmarks, including reduced grey matter volume and increased amyloid burden. Although effect sizes are generally moderate, these associations support a biologically meaningful link between systemic inflammaging and CNS dysfunction, reinforcing the translational relevance of peripheral immune biomarkers [157,158,159].

## 4. Adaptive Immune Aging and Loss of Immune Homeostasis in the Aging Brain

Aging profoundly reshapes the adaptive immune compartment, affecting lymphocyte development, repertoire diversity, functional plasticity, and regulatory balance [31]. These alterations originate in hematopoietic stem cells and progressively propagate across lymphoid organs and peripheral compartments. Within the CNS, age-related adaptive immune alterations acquire CNS-specific features that contribute to neuroinflammation and are associated with impaired brain function. Adaptive immunosenescence thus extends beyond systemic immune decline, actively shaping CNS immune remodeling and contributing to neurodegenerative vulnerability.

**Figure 2:**
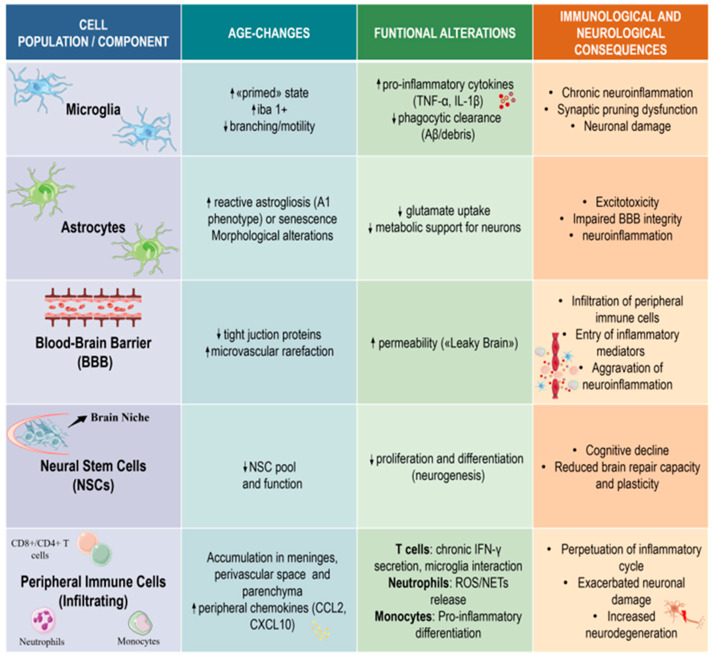
Immunosenescence in the brain: cellular and functional changes. The figure illustrates the key cellular and structural components of neuroinflammation, highlighting their phenotypic and functional shifts and the resulting neurological consequences. Microglia transition to a “primed” state, characterized by reduced motility and increased secretion of pro-inflammatory cytokines, leading to impaired synaptic pruning and chronic neuroinflammation. Astrocytes exhibit reactive astrogliosis (A1 phenotype), leading to diminished glutamate uptake and metabolic support, which contribute to excitotoxicity and BBB dysfunction. The BBB shows reduced tight junction protein expression and microvascular rarefaction, thereby increasing permeability and facilitating peripheral immune cell infiltration. NSCs experience depletion of the progenitor pool and impaired neurogenesis, which underlie cognitive decline and reduced neuroplasticity. Infiltrating peripheral immune cells exacerbate the inflammatory cycle and pro-inflammatory differentiation, ultimately contributing to neuronal damage and neurodegeneration. ↑ indicates “increase” or “upregulation”; ↓ indicates “decrease” or “downregulation”. The references corresponding to the studies originally reported in the last column of Figure 2 are indicated here: Row 1 [30,106,114,115,160]; Row 2 [128,132,133,134,135,136]; Row 3 [143,144,145,146,147,148]; Row 4 [96,97]; Row 5 [16,161,162,163,164,165].

The main cellular and functional changes that occur during immunosenescence in the brain are outlined in Figure 2. 

### 4.1. T Cells and Regulatory T Cells in CNS Aging

Age-related remodeling of the T cell compartment extends into the CNS, where adaptive immune cells contribute to tissue surveillance and neuroimmune crosstalk. In the meningeal compartments surrounding dural sinuses, T cells survey cerebrospinal fluid-derived antigens through local antigen-presenting cells and initiate adaptive immune responses [165]. Moreover, clonally expanded CNS antigen-reactive T cells are detected in the brain parenchyma and neurogenic niches [166].

With aging, hallmark features of T cell immunosenescence emerge, including reduced naïve T cell output, contraction of TCR diversity, and expansion of highly differentiated memory and CD28− subsets [38,167]. This shift toward terminal differentiation reduces adaptive flexibility and promotes a pro-inflammatory dominance. Even during physiological aging, senescent T cells infiltrate the CNS parenchyma and meninges, establishing a ‘neuro-inflammaging’ milieu that compromises glial and neuronal homeostasis, thereby increasing CNS vulnerability to proteotoxic stress and injury [92,168].

Senescent-like CD8+ T cells accumulate with age and are associated with cognitive decline [169]. Their recruitment to the aging brain, partly mediated by CXCL10/CXCR3 signaling, promotes axonal degeneration and impaired myelination in experimental models [92,168]. CD8+ T cells produce high levels of IFNγ [161], establishing an inflammatory niche that compromises oligodendrocyte and neuronal homeostasis. Brain CD8+ tissue-resident memory (TRM) T cells accumulate with age and represent a major source of IFNγ, inducing IFN-responsive transcriptional programs in oligodendrocytes and microglia [161]. Genetic ablation of CD8+ T cells rescues age-associated oligodendrocyte loss, whereas enhanced CD8+ T cell activity or exogenous IFNγ accelerates glial activation and neurodegeneration [161]. IFNγ further impairs neural stem cell proliferation [166], promotes oligodendrocyte injury [170], induces axonal alterations [171] and amplifies microglia-mediated damage [161].

Regulatory T cells (Tregs) normally maintain CNS immune equilibrium, but aging impairs their frequency and function [56,172], weakening their control over effector T cell activation, sustaining inflammatory signaling within the CNS, and amplifying neuronal vulnerability [162,173]. Impaired Treg-mediated restraint exacerbates the pro-inflammatory imbalance characteristic of immunosenescence.

These age-associated changes in effector and regulatory T cell compartments reshape CNS immune dynamics. Persistent cytokine production, antigen-driven clonal expansion, tissue residency, and impaired regulation position T cells as active contributors to neuroinflammation and age-related neurodegenerative susceptibility rather than passive correlates of brain aging.

### 4.2. B Cell Senescence in the CNS

Within the CNS, age-related B cell alterations contribute to a dysregulated neuroimmune environment that is associated with chronic neuroinflammation [174]. B cells influence CNS pathology by modulating microglial activation, antibody and cytokine production [174].

Aging is characterized by the expansion of pro-inflammatory double-negative B cell subsets (IgD-CD27-) with a CD95+CD21-CD11c+ phenotype and spontaneous T-bet expression [175]. These age-associated B cell alterations provide a mechanistic link between systemic immunosenescence and CNS neuroinflammatory remodeling. These cells secrete inflammatory mediators and autoantibodies, respond poorly to vaccination and may reinforce systemic and potentially CNS-directed immune activation, representing a hallmark of B cell immunosenescence [175].

Experimental models support a functional role for B cells in neuroinflammation. In AD, non-Aβ-specific IgG antibodies can enhance amyloid-β plaque dynamics by promoting microglial phagocytosis through Fc receptor-mediated pathways [176]. Depletion of adaptive lymphocytes in Rag-5xfAD mice worsens pathology, whereas their restoration mitigates neuroinflammation [176], indicating that B cells exert both protective and pathogenic effects in the aging brain.

B cells can also produce neurotrophic factors, such as brain-derived neurotrophic factor (BDNF), glial cell line-derived neurotrophic factor (GDNF) and neurotrophin-3 (NT-3) [174], which support neuronal survival. However, immunosenescence-associated reductions in naïve B cells, impaired clonal expansion, and qualitative and quantitative antibody dysfunction [177] may compromise this neuroprotective potential, shifting the balance toward neurodegeneration.

Age-related immune remodeling further alters CNS immune-glial crosstalk. Primed microglia display exaggerated cytokine production [178,179,180], amplifying the effects of B-cell-derived inflammatory mediators. Increased BBB permeability facilitates infiltration of peripheral immune cells into the CNS [178], enhancing interactions between senescent B cells and resident glia. In parallel, chronic low-grade inflammation and SASP-producing senescent cells sustain neuroinflammatory signaling [19,181].

Bidirectional communication between bone marrow-derived immune cells and CNS-resident glia highlights how B cell senescence feeds into a self-sustaining neuroinflammatory network, linking peripheral immunosenescence to central pathology [182,183].

Collectively, B cell immunosenescence shifts neuroimmune surveillance toward persistent inflammatory activation. Through altered subset composition, dysfunctional antibody responses, and impaired regulatory capacity, senescent B cells are thought to reinforce self-sustaining neuroinflammatory circuits in the aging brain [174].

## 5. Neurodegenerative Diseases in the Context of Immunosenescence

The cellular and molecular mechanisms outlined in Figure 1 and Figure 2 underpin immunosenescence-driven pathogenesis across major neurodegenerative diseases. These findings support a significant contribution of immune aging processes rather than a purely secondary epiphenomenon in NDDs. However, current evidence supports a bidirectional model in which immunosenescence and neurodegeneration mutually reinforce each other. While causal relationships are well supported in experimental models (e.g., immune cell depletion, senolytic interventions), human data are predominantly associative, highlighting the need for cautious interpretation of causality [13,14]. Across AD, PD, ALS and MS, immunosenescence and inflammaging synergistically contribute to disease onset, progression, and therapeutic response, primarily through senescent microglia and astrocytes. These cells secrete pro-inflammatory SASP mediators, disrupting neuronal homeostasis, promoting synaptic dysfunction and accelerating neurodegeneration [184,185,186]. These processes, combined with the above-described oxidative stress, mitochondrial dysfunction, and impaired proteostasis, establish a chronic neuroinflammatory environment.

A self-perpetuating loop emerges in which aged innate and adaptive immune cells fail to efficiently clear pathological proteins, while accumulating misfolded proteins (Aβ, Tau, and α-synuclein). These processes further accelerate immune cell senescence, reinforce systemic inflammaging, and consolidate a feed-forward circuit linking peripheral and CNS immune aging [187,188,189,190]. Thus, immunosenescence emerges as a central component contributing to NDD amplification rather than merely a correlate of aging.

### 5.1. Alzheimer’s Disease

AD exemplifies the interplay between immunosenescence, chronic inflammation and neurodegeneration. Microglia, as CNS innate immune effectors, physiologically clear soluble and aggregated Aβ via receptor-mediated phagocytosis and pinocytosis [191,192,193]. This clearance process involves several key receptors, including Toll-like receptor 4 (TLR4), and CX3CR1, which coordinate the recognition and internalization of amyloid fibrils [193,194].

Aging impairs microglial clearance, promoting Aβ accumulation and plaque formation. Concurrent oxidative stress, mitochondrial dysfunction, and defective mitophagy sustain the release of mitochondrial DAMPs, including mtDNA, activating the cGAS–STING and NLRP3 inflammasome pathways and driving persistent neuroinflammaging through IL-1β and IL-18 production. Aβ binding to pattern recognition receptors further amplifies inflammatory signaling in experimental models, thereby exacerbating neuronal injury. Senescent microglia undergo heterochromatin remodeling, stabilizing the SASP and reinforcing neuroinflammaging. Peripheral immune aging is also evident in AD patients, who show telomere shortening and an increase in senescent CD8+ effector memory T cells, including CD45RA+ TEMRA cells, which correlate negatively with cognitive performance [195,196,197]. These findings suggest that systemic inflammaging may lower the threshold for CNS vulnerability and potentially amplify local neuroinflammaging processes.

Astrocyte senescence amplifies AD pathology by inducing excitotoxicity and sustaining neuroinflammation [128]. Tau oligomers trigger astrocyte senescence through HMGB1 release and SASP-mediated paracrine signaling, while Aβ, oxidative stress and IL-1β further reinforce this phenotype in model systems [129,130]. YAP inactivation contributes to premature astrocyte senescence, SASP expression, and loss of neuroprotective functions [131].

### 5.2. Parkinson’s Disease

PD is characterized by the loss of dopaminergic neurons and by α-synuclein aggregation in the context of systemic and CNS immune aging. Age-related immunosenescence involves reduced numbers of naïve CD4+ and CD8+ T cells, naïve B cells, NK cells, and plasma cells, with expansion of late-differentiated and senescent phenotypes [198,199,200,201]. This decline correlates with increased PD susceptibility and reduced striatal dopamine transporter levels [202,203,204]. PD patients exhibit increased late-differentiated senescent CD4+ and CD8+ T cells, which are inversely associated with age at disease onset [198], as well as elevated neutrophil and monocyte counts, greater motor impairment, and dopaminergic neuron loss [205]. PD is also associated with atypical CD8+ T cell senescence, characterized by reduced p16INK4A expression [206,207].

In the CNS, α-synuclein has been shown to promote glial senescence and defective proteostasis. Aged microglia exhibit impaired autophagy-lysosome function and increased accumulation of α-synuclein in aged mice [208]. Monocytes from elderly individuals similarly show reduced phagocytic capacity [209]. Impaired mitophagy enhances ROS production and inflammasome activation, reinforcing neuroinflammaging. Senescent microglia and astrocytes amplify α-synuclein aggregation through SASP-mediated inflammation [210,211]. Activation of the cGAS-STING pathway in senescent astrocytes promotes neurotoxicity, and astrocyte-specific cGAS depletion attenuates senescence and neurodegeneration in PD models [211].

Consistent with age-associated BBB vulnerability discussed above, the infiltration of activated monocytes and T cells into the brain exacerbates neuroinflammation and neuronal injury [164]. T cell infiltration is observed in PD brains, and circulating T cells from PD patients display altered dopamine metabolism and mitochondrial dysfunction, supporting bidirectional neuroimmune crosstalk [212,213,214].

### 5.3. Amyotrophic Lateral Sclerosis

ALS is characterized by progressive degeneration of upper and lower motor neurons, with age as the strongest risk factor. Emerging evidence implicates immunosenescence as a key contributor to disease-associated neuroinflammation and progression [215,216,217,218]. Peripheral immune profiling reveals an increase in senescent and late-memory T and B cells, particularly in rapidly progressing patients [218]. Experimental data indicate that CD4+ T cell senescence is associated with neuroinflammation and accelerates motor neuron degeneration, linking adaptive immunosenescence to systemic inflammaging and CNS pathology [219].

In the CNS, senescence markers are detected in microglia and astrocytes in the spinal cord and cortex of ALS patients and animal models [220,221]. These cells exhibit sustained pro-inflammatory activation, increased ROS production and impaired neuroprotective functions.

Neuroinflammation evolves dynamically, with early protective microglial responses shifting toward a neurotoxic phenotype as it progresses [222,223]. Neuronal injury releases misfolded proteins and aberrant peptides that activate innate immunity and may be presented via MHC class I, triggering cytotoxic CD8+ T cell-mediated neuronal injury under inflammatory and oxidative conditions [224,225,226]. Systemically, ALS patients display chronic low-grade inflammation, elevated pro-inflammatory cytokines and impaired immune regulation [216,227,228]. Reduced Treg levels correlate with faster disease progression, highlighting the contribution of immune imbalance to neurodegeneration [227,228]. Together, immune system-driven inflammaging emerges as a central amplifier of neuroinflammatory toxicity in ALS.

### 5.4. Multiple Sclerosis

MS represents a paradigmatic context in which immunosenescence intersects with chronic autoimmune neuroinflammation and neurodegeneration. Premature immune aging is present in MS patients and includes thymic involution, telomere shortening, expansion of late-differentiated CD8+ T cells, accumulation of age-associated B cells, and accelerated epigenetic aging of peripheral immune cells [38,229,230,231,232]. These alterations are associated with increased disability, reduced response to disease-modifying therapies and higher risk of transition to progressive MS [233].

Within the CNS, senescent microglia exhibit impaired myelin debris clearance and sustained production of inflammatory mediators, limiting remyelination and exacerbating axonal damage [185,234]. Aging macrophages similarly display reduced regenerative efficiency [235]. These defects promote a chronic, compartmentalized neuroinflammatory state within the CNS that drives progression even in the absence of overt BBB disruption.

Experimental models demonstrate that immunosenescence skews the immune response toward pro-inflammatory Th1 and Th17 responses, reduces Treg and NK cell function, and intensifies neurodegeneration in aged hosts [234,236,237]. MS pathogenesis thus involves both acute immune infiltration during relapses and a slower accumulation of late-differentiated immune cells in meningeal and perivascular niches, sustaining chronic neuroinflammation [238]. In progressive MS, microglial activation, oxidative injury and mitochondrial dysfunction become dominant drivers of disability progression and therapeutic resistance [239].

Although senolytic approaches show promise in other neurodegenerative settings, limited efficacy in autoimmune models suggests that targeting senescence alone may be insufficient [240]. Effective strategies will likely require stage-specific modulation of both systemic inflammaging and CNS-compartmentalized neuroinflammaging.

## 6. Emerging Therapeutic Strategies Targeting Immune Aging and Neurodegeneration

The growing recognition of immunosenescence and inflammaging as central drivers of neurodegeneration has sparked intense interest in therapeutic strategies to restore immune homeostasis, attenuate chronic inflammation, and selectively target senescent cells. However, despite promising preclinical evidence, translating these interventions into effective clinical practice has proven challenging, particularly in humans [241]. This gap reflects the complexity of immune aging, the heterogeneity of neurodegenerative diseases, and the critical importance of timing, tissue specificity, and immune compartmentalization. Importantly, most of these approaches remain at preclinical or early clinical stages, and robust evidence of disease-modifying efficacy in humans is still limited.

### 6.1. Senotherapeutics: Senolytics and Senomorphics

Senotherapeutics encompass two major classes of interventions: senolytics, which selectively eliminate senescent cells, and senomorphics, which modulate senescent phenotypes without inducing cell death. Senolytic strategies have attracted particular attention for their ability to reduce senescent cell burden and attenuate SASP-driven inflammation in preclinical models [18,242,243].

Several senolytic compounds, including dasatinib, quercetin, navitoclax (ABT263) and ABT-737, have demonstrated efficacy in eliminating senescent immune and glial cells in preclinical animal and non-human primate models. At the same time, pharmacological inhibition of anti-apoptotic BCL-2 family proteins using ABT-737 or ABT263 effectively removes senescent microglia and astrocytes, leading to reduced neuroinflammation and improved cognitive performance in mouse models of AD and Tauopathy [9,244,245,246].

Importantly, recent studies have demonstrated that navitoclax can penetrate the BBB, reduce senescence and SASP biomarkers in cerebrospinal fluid and improve biomarkers of neuroinflammation and synaptic dysfunction in aged non-human primates, with an acceptable safety profile [246]. Nevertheless, translation to human disease remains uncertain.

Phase I clinical trial of dasatinib plus quercetin in early-stage symptomatic AD patients did not demonstrate significant cognitive improvement, despite acceptable tolerability [247,248]. Moreover, the dasatinib-quercetin combination failed to ameliorate disease severity in experimental autoimmune encephalomyelitis in mice, a widely used murine model of multiple sclerosis. This lack of efficacy suggests that senescent cell elimination alone may be insufficient in diseases characterized by persistent immune activation and autoimmunity [240].

These mixed outcomes highlight several unresolved challenges. First, senescent cells may exert context-dependent roles, including tissue repair and immune regulation, raising concerns about indiscriminate depletion. Second, systemic senolysis may impair immune surveillance, increasing susceptibility to infections and malignancies. Third, the optimal therapeutic window remains unclear: senolytics may be effective in early or pre-symptomatic stages, but less so once irreversible neurodegeneration has occurred.

Senomorphics offer a complementary strategy by suppressing SASP and inflammatory signaling without eliminating senescent cells. Targeting pathways such as mTOR, NF-κB, p38 MAPK, and cGAS-STING may attenuate inflammaging while preserving essential cellular functions [30,44,211]. However, the long-term consequences of chronic senomorphic modulation on immune competence and CNS resilience remain poorly understood. Notably, the majority of senolytic evidence derives from animal models and clinical data remains scarce and inconclusive. Existing human studies are limited in size and duration, and no robust, conclusive evidence of clinical efficacy in NDDs has yet been established [249].

### 6.2. Immunomodulation and Immune Rejuvenation

Beyond senescence-targeted approaches, strategies to restore immune balance and rejuvenate immune function have shown promise in experimental settings. In AD models, transplantation of young splenocytes enhances amyloid clearance, reduces astrogliosis, and improves cognitive performance, suggesting that restoring youthful immune signaling may counteract neurodegenerative pathology [250]. However, translating these findings into human disease remains challenging, highlighting the need for a better understanding of timing, compartmental specificity, and immune-brain communication. Similarly, inhibition of immune checkpoints such as PD-1/PD-L1 has been proposed to enhance immunosurveillance and the clearance of pathological proteins in model systems, although concerns regarding autoimmunity and neurotoxicity persist [196,251,252,253].

However, immune rejuvenation strategies face substantial translational barriers. Systemic immune activation risks exacerbating neuroinflammation, particularly in the context of BBB dysfunction and CNS immune cell priming. Moreover, peripheral immune interventions may fail to adequately target CNS-resident senescent cells, underscoring the importance of compartment-specific therapeutic design.

### 6.3. Immunoceuticals and Nutraceuticals as Modulators of Immune Aging and Neurodegeneration

Dietary interventions have emerged as powerful modulators of immune aging and inflammaging, influencing immune cell metabolism, inflammatory tone, and systemic resilience. Both nutrient-specific components and broader dietary patterns shape immune function across the lifespan, with particularly relevant effects in older adults, where chronic low-grade inflammation and metabolic dysregulation accelerate immunosenescence [18]. Rather than acting as disease-modifying agents per se, immunoceuticals and nutraceuticals are increasingly viewed as long-term modulators capable of delaying immune aging, attenuating inflammaging and enhancing responsiveness to other therapeutic interventions. Importantly, these systemic effects may translate into neuroprotective benefits; by attenuating peripheral inflammaging and modulating the gut–brain axis, immunoceuticals can reduce microglial activation, preserve BBB integrity and mitigate pro-inflammatory signaling that drives neuronal decay [254,255].

Although supported by epidemiological and small-scale clinical studies, immunoceuticals should be regarded as modulatory rather than disease-modifying interventions, with heterogeneous effects across populations and limited mechanistic validation in humans.

#### 6.3.1. Polyphenols and Related Bioactive Compounds

Polyphenols (e.g., resveratrol, curcumin, and flavonoids) exert pleiotropic antioxidant and anti-inflammatory effects relevant to immune aging [256,257]. Preclinical studies demonstrate their ability to modulate both innate and adaptive immunity. In murine models, resveratrol enhances T cell proliferation, increases NK cell activity, and reduces pro-inflammatory cytokines such as IL-6 and TNF-α [77,258]. Polyphenols also reduce oxidative stress, improve macrophage function, and modulate NF-κB and Nrf2 signaling, key pathways of immune regulation [259], reducing IL-1β and IL-18 release and attenuating SASP-driven inflammation [29,260].

Resveratrol and related stilbenes stimulate SIRT1 signaling and PGC-1α–mediated mitochondrial biogenesis, thereby improving lymphocyte bioenergetics and sustaining T cell function during aging [261,262].

Polyphenols can cross the BBB [263,264] and exert neuroprotective effects by modulating neuronal signaling pathways and scavenging reactive oxygen and nitrogen species [256,257,265,266]. Limited human evidence supports these findings.

Flavonoid-rich foods enhance NK cell activity and reduce inflammatory markers in older adults [267,268], while resveratrol supplementation has been associated with reduced CRP and IL-6 and improved T cell proliferation. Polyphenols may also induce epigenetic remodeling of immune cells [269], and their activity may partly depend on gut microbiota-derived metabolites, given their limited bioavailability [98].

Overall, dietary polyphenols may modulate both systemic immunosenescence and CNS vulnerability, potentially attenuating mechanisms linking chronic inflammation to neurodegeneration.

#### 6.3.2. Omega-3 Polyunsaturated Fatty Acids (PUFAs)

Omega-3 polyunsaturated fatty acids (PUFAs), particularly eicosapentaenoic acid (EPA) and docosahexaenoic acid (DHA), are well-characterized nutrients that modulate the immune system, particularly in the context of immune aging. They are crucial structural components of neuronal membranes and play a vital role in maintaining synaptic plasticity, neurotransmission, and membrane fluidity. Omega-3 PUFAs reduce pro-inflammatory cytokine production by activating peroxisome proliferator-activated receptors and inhibiting NF-κB [270]. Additionally, they serve as precursors for specialized pro-resolving mediators (SPMs), such as resolvins and protectins, which actively drive resolution of inflammation rather than merely suppressing its initiation [270]. Interestingly, by improving membrane fluidity and receptor signaling, omega-3 PUFAs enhance macrophage and T cell activity, supporting both innate and adaptive immune responses [271].

Additionally, preclinical evidence highlights the neuroprotective potential of omega-3 fatty acids. In animal models of neurodegeneration, DHA supplementation reduces amyloid-β accumulation, attenuates Tau pathology and improves synaptic function. In APP transgenic models, DHA-enriched diets consistently decrease Aβ levels and mitigate neuroinflammatory responses [272,273,274,275,276,277,278].

Further preclinical studies show that DHA, along with EPA, exerts anti-inflammatory, antioxidant, and anti-apoptotic effects, attenuating the production of pro-inflammatory cytokines such as IL-6 and TNF-α [279,280,281]. In addition, higher intake of n-3 PUFAs has been associated with reduced PD risk in MPTP-induced mouse models; these lipids prevent dopaminergic neuronal loss and preserve striatal dopamine levels [279,282,283,284,285,286,287,288].

Epidemiological and clinical studies provide additional support for preclinical observations. Higher dietary intake of n-3 PUFAs has been associated with a reduced risk of cognitive decline and dementia, and some clinical trials in individuals with mild-to-moderate AD have reported modest cognitive improvements following DHA-containing interventions [289,290].

Additionally, higher brain DHA levels have been associated with improved cognitive outcomes through modulation of neurotransmitter release, gene expression and neuroinflammatory signaling [278,291]. Omega-3 supplementation in older adults has also been shown to reduce circulating inflammatory markers, including CRP, highlighting their systemic anti-inflammatory effects [292,293].

Collectively, these findings suggest that omega-3 PUFAs may help maintain brain homeostasis by modulating both systemic and neuroinflammatory processes. However, they are better considered regulators of inflammatory tone rather than stand-alone disease-modifying therapies [294].

### 6.4. Caloric Restriction Mimetics and Autophagy-Based Interventions

Caloric restriction (CR) is one of the most robust gerotherapeutic interventions, extending lifespan and healthspan across multiple species and conferring immune and neuroprotective benefits in both animals and humans [295]. CR induces beneficial changes in gut microbiota composition, increases naïve T cell populations, reduces exhausted immune cells and improves immune function in aged organisms [296,297,298]. However, long-term adherence to CR or intermittent fasting is challenging, motivating the development of caloric restriction mimetics (CRMs).

CRMs are natural or pharmacological compounds that reproduce key molecular effects of CR, particularly activation of autophagy and mitochondrial quality control pathways [296]. CRMs regulate redox homeostasis by activating the Nrf2 pathway, enhancing antioxidant defenses by dissociating Nrf2 from Keap1 and transcriptionally activating antioxidant response elements [295,299,300]. Concurrently, CRMs attenuate mitochondrial dysfunction, inhibit excessive ROS production, and modulate redox-sensitive signaling pathways such as PI3K/Akt and MAPK, thereby promoting neuronal survival and immune resilience [295].

By restoring mitochondrial metabolism and autophagic flux, CR and CRMs decrease systemic inflammation, safeguard intestinal barrier integrity, and reduce both inflammaging and neuroinflammation. Compounds such as NAD+ precursors, metformin, spermidine, rapamycin, and resveratrol influence autophagy, mitochondrial function, and inflammatory pathways, collectively improving resistance to age-related immune and neurodegenerative disorders. Nonetheless, the pleiotropic nature of these interventions requires careful consideration of dosage, timing, and long-term safety.

### 6.5. Modulation of the Gut–Brain–Immune Axis

Bidirectional communication between the gastrointestinal tract and the central nervous system, the gut–brain–immune axis, plays a critical role in immune development, homeostasis and aging. Gut microbiota influence CNS function through regulation of the hypothalamic–pituitary–adrenal (HPA) axis, modulation of cortisol release and production of neurotransmitters, neuropeptides, endocrine hormones, and immunomodulatory molecules [98,301,302,303]. These signals shape microglial activation states, cytokine release, and recruitment of peripheral immune cells to the brain.

The gut microbiota is essential for the development and maintenance of the gut-associated lymphoid tissue (GALT), which harbors approximately 70% of the body’s circulating lymphocytes [304]. Aging is associated with pronounced alterations in microbiome composition, characterized by reduced diversity and the expansion of pro-inflammatory taxa [301]. The aged microbiome promotes production of pro-inflammatory cytokines (e.g., TNF-α, IL-2, IL-6, IL-8, IFN-γ), thereby exacerbating systemic inflammation and accelerating immunosenescence [301,302].

Experimental studies demonstrate that fecal microbiota transplantation (FMT) from young donors rejuvenates aged HSCs, promotes lymphoid differentiation, suppresses myeloid skewing, and activates anti-aging FOXO signaling pathways [305]. Microbiota-driven signaling also regulates neutrophil aging via TLRs and MyD88-dependent pathways, thereby reducing inflammation-related organ damage [306]. In the CNS, microbiota-induced transcriptional remodeling of microglia contributes to age-associated phenotypes, while microbiota depletion attenuates oxidative stress and mitochondrial dysfunction in aged microglia of mice [98]. Commensal bacteria further drive the accumulation of senescent and disease-associated microglia during aging [108].

Probiotic interventions similarly show promise: *Lactobacillus plantarum* GKM3 enhances memory retention, extends longevity, and reduces oxidative stress in senescence-accelerated mice [307], while supplementation with *Bifidobacterium longum* Bar33 and *Lactobacillus helveticus* Bar13 increases naïve T cells, regulatory T cells, B cells and NK cell activity, while reducing memory T cells, indicating robust anti-IS effects [308].

In neurodegenerative contexts, FMT has been reported to improve gastrointestinal and autonomic symptoms in PD and increase microbial diversity [232]. Recent clinical evidence has begun to clarify these effects. For instance, the GUT-PARFECT trial (NCT03808389), a randomized, placebo-controlled study, reported that a single healthy-donor FMT led to sustained improvements in motor symptoms in patients with early-stage PD over 12 months [309]. However, larger trials have not consistently demonstrated a significant impact on disease progression [310]. In AD, clinical data remain limited. Trials such as the AMBITION study (NCT03998423) have primarily assessed safety and feasibility, showing that FMT can modulate microbial composition and systemic inflammatory biomarkers. However, its efficacy in improving cognitive outcomes remains to be established in large, controlled studies [311]. These mixed outcomes underscore the complexity and disease specificity of microbiota-immune-brain interactions.

Microbiota-targeted strategies, including probiotics, prebiotics, postbiotics, dietary interventions and FMT, offer promising avenues to restore gut homeostasis and mitigate age-related immune dysfunction [243,312]. Commensal bacteria interact with host immune cells via microbial-associated molecular patterns (MAMPs), which activate TLRs and shape immune maturation and function [313,314]. While probiotic development holds significant therapeutic potential, responses are highly individualized, and precision approaches will be required to maximize efficacy.

## 7. Novel Drug Delivery Systems Targeting Immune Aging in the CNS

One of the major barriers to effective treatment of neurodegenerative diseases is limited drug delivery across the BBB. Nanotechnology offers transformative potential to overcome these constraints by enabling targeted transport, cell-specific delivery, and stimulus-responsive release of therapeutic payloads [315,316].

Nanoparticles (NPs), typically 1–100 nm in size, can be engineered from metals, polymers, or carbon-based materials and tailored for BBB penetration, biocompatibility, and controlled release [317,318,319,320]. Recent advances demonstrate that nanoparticle-based delivery of p16INK4A siRNA attenuates Aβ deposition and microglial senescence surrounding plaques, improving cognitive performance in AD animal models [321]. In PD models, chiral gold NPs carrying anti-B2MG and anti-DCR2 antibodies selectively remove senescent microglia and reduce CSF α-synuclein levels [322].

Additional nanotechnological approaches include lipid-based carriers and polymeric NPs that reduce neuroinflammation and pathological protein aggregation. Phosphatidic acid-, cardiolipin-, PEG-, and PLGA-based liposomes promote clearance of Aβ aggregates and attenuate neuroinflammation in AD models [322,323,324,325,326]. Gold NPs functionalized with chiral peptide inhibitors reduce Aβ42 fibrillization and improve spatial learning and memory in vivo [327]. Antioxidant-loaded nanocarriers further enhance CNS delivery and ROS scavenging, rescuing redox imbalance and restoring antioxidant gene expression in PD models [327].

Despite strong preclinical promise, safety, biodistribution, long-term toxicity, and immunogenicity remain key challenges for clinical translation of nanoparticle-based therapies. A summary of the discussed therapeutic strategies and novel drug delivery methods is provided in Table 1.

## 8. Translational Challenges and Unresolved Gaps

Despite compelling evidence that immune aging is a key driver of neurodegenerative diseases, several conceptual and translational challenges remain. A major limitation is the lack of validated, disease-relevant biomarkers that reliably capture immunosenescence and inflammaging in humans. Immune aging is a multidimensional process encompassing cellular senescence, altered immune repertoire diversity, metabolic dysfunction and chronic inflammatory signaling, yet most clinical studies rely on isolated markers or systemic inflammatory readouts. Future efforts will require integrated biomarker frameworks that combine cellular senescence markers (e.g., p16INK4A expression in immune subsets), immune repertoire remodeling, epigenetic aging signatures, mitochondrial stress indicators, and circulating inflammatory and SASP-related profiles to define immune aging states predictive of neurodegenerative risk and progression.

Another critical challenge lies in bridging mechanistic insights from basic immunology and neurobiology with clinical trial design. Preclinical models have convincingly demonstrated that immunosenescence and inflammaging actively shape glial dysfunction, BBB integrity, and neuronal vulnerability. However, most clinical interventions are initiated at symptomatic stages, long after immune-driven neuroinflammatory loops are established. This temporal mismatch likely contributes to the limited efficacy of immune-modulating and senescence-targeting therapies in human neurodegenerative diseases. Translational strategies must therefore prioritize early intervention windows, stratification of patients by immune-aging phenotypes, and a clearer distinction between systemic and CNS-compartment-specific immune dysfunction.

A further emerging concept is the necessity of multimodal intervention strategies. Pharmacological approaches targeting senescent cells, inflammatory pathways, or immune checkpoints are unlikely to be sufficient in isolation, given the systemic and self-reinforcing nature of immune aging. Lifestyle factors such as physical activity, diet, sleep quality, and circadian alignment exert profound effects on immune metabolism, inflammatory tone, mitochondrial function and gut microbiota composition. Integrating non-pharmacological interventions with targeted immune-modulating therapies may be essential to effectively reduce the effects of inflammaging and maintain neuroimmune homeostasis during aging.

Finally, immunosenescence should be reframed not only as a therapeutic target but also as a modifiable determinant of healthspan and cognitive resilience. In this context, immune aging should be viewed not merely as a consequence of aging but as a central, modifiable driver of neurodegenerative vulnerability. Future strategies should therefore focus on early identification of immunosenescence phenotypes, integrating lifestyle factors with pharmacological interventions, and developing precision approaches to restore neuroimmune homeostasis before irreparable neuronal damage occurs.

Such prevention-focused, immune-informed strategies hold substantial promise for reducing the burden of neurodegenerative diseases in aging populations and for translating advances in immune geroscience into tangible clinical benefit.

## Figures and Tables

**Table 1:** Summary of therapeutic strategies and nanoparticle-based delivery targeting immunosenescence and inflammaging in neurodegeneration.

Strategy	Mechanism of Action	Supporting Evidence	Target	Limitations	Key References
Senolytics	Selective elimination of senescent cells and reduction of SASP	Reduced neuroinflammation and improved cognitive performance in animal models; no significant cognitive benefit observed in AD patients	Senescent cells	Context-dependent effects; potential impairment of physiological immune functions	[9,18,240,242,243,244,245,246,247]
Senomorphics	Suppression of SASP and inflammatory signaling pathways (mTOR, NF-κB, p38 MAPK, cGAS-STING)	Attenuation of inflammaging and modulation of inflammatory pathways in preclinical models	Senescent cells	Long-term effects on immune competence remain unclear	[30,44,211]
Immune rejuvenation	Restoration of immune cell function and signaling	Enhanced amyloid clearance, reduced astrogliosis, and improved cognitive performance in AD models	Peripheral immune system	Limited clinical evidence; translational feasibility remains uncertain	[250]
Checkpoint modulation	Enhancement of immune surveillance and clearance of pathological proteins	Preclinical evidence suggests improved clearance of toxic protein aggregates	T cells	Risk of autoimmunity and neurotoxicity	[196,251,252,253]
Microbiome-based therapies	Modulation of the gut–brain–immune axis	Rejuvenation of hematopoietic stem cells and immune function in models; improved gut health in PD patients, with inconsistent neurological outcomes	Gut–immune axis; CNS immune cells	High inter-individual variability; lack of standardization	[232,305,306,307,308,310]
Immunoceuticals Nutraceuticals	Anti-inflammatory, antioxidant, and metabolic modulation	Reduction of inflammatory markers (e.g., IL-6, CRP), improved immune cell function, modest cognitive benefits	Immune system; CNS	Primarily modulatory effects; not disease-modifying	[256,257,258,259,260,278,289,290,291,292,293]
Caloric restriction mimetics	Activation of autophagy and mitochondrial function	Improved immune function, reduced inflammation, and enhanced metabolic regulation in preclinical studies	Immune metabolism	Optimal dosing, safety, and long-term effects require further investigations	[295,296,297,298,299,300]
Nanoparticle-based delivery	Targeted delivery across the blood–brain barrier (BBB)	Reduced Aβ deposition, decreased microglial senescence, and improved cognitive performance in models	BBB; CNS cells	Safety, biodistribution, and toxicity are not fully established	[315,316,317,318,319,320,321,322,323,324,325,326,327]

## Data Availability

No new data were created or analyzed in this study.

## Abbreviations

The following abbreviations are used in this manuscript:

AD	Alzheimer’s Disease
ALS	Amyotrophic Lateral Sclerosis
Aβ	Amyloid-β
BBB	Blood–brain barrier
BDNF	Brain-derived neurotrophic factor
CNS	Central nervous system
CR	Caloric restriction
CRMs	Caloric restriction mimetics
CRP	C-reactive protein
DAMPs	Damage-associated molecular patterns
DHA	Docosahexaenoic acid
EPA	Eicosapentaenoic acid
ERCC1	Excision repair 1, non-catalytic subunit
GDNF	Glial cell line-derived neurotrophic factor
HPA	Hypothalamic–pituitary–adrenal axis
MS	Multiple sclerosis
NDDs	Neurodegenerative Diseases
NK	Natural killer
NPs	Nanoparticles
NT-3	Neurotrophin-3
PD	Parkinson’s Disease
PUFAs	Polyunsaturated fatty acids
TCR	T cell receptor
TLRs	Toll-like receptors
Tregs	Regulatory T cells
TREM2	Triggering receptor expressed on myeloid cells 2
TRM	Tissue-resident memory T cells
YAP	Yes-associated protein
